# Supplementary material for: Kidney Transplant Recipients with Acute Antibody-Mediated Rejection Show Altered Levels of Matrix Metalloproteinases and Their Inhibitors: Evaluation of Circulating MMP and TIMP Profiles
Source: Int J Mol Sci. 2025 Jun 23;26(13):6011. doi: 10.3390/ijms26136011 (PMC12250063; doi:10.3390/ijms26136011)
Supplement: Supplementary file 1 [file ijms-26-06011-s001.zip › ijms-3616493-supplementary.pdf]

Supplementary Materials:

Table S1. Comparison of plasma biomarkers between patients with and without rejection, stratified by age group (<40 vs ≥40 years).

Age	Biomarker	NR (Mediana [IQR])	ABMR (Mediana [IQR])	n (NR / ABMR)	*P* value
<40	MMP-1	885.8 (827.39–1197.03)	1722 (1080.31–3405.17)	3/10	0.16
	MMP-2	1310 (1061.80–1858.50)	3426 (2095.95–3948.75)	3/10	0.08
	MMP-3	970.1(827.39–1197.03)	2510 (1080.31–3405.17)	3/10	0.22
	TIMP-3	987 (112.66–1078.91)	504.6 (413.48–841.91)	3/10	0.66
>40	MMP-1	983 (807.80–992.76)	1100 (846.84–2782.61)	9/5	0.17
	MMP-2	2655 (1708.65–1421.50)	3108 (2560.10–3751.40)	9/5	0.14
	MMP-3	583.5 (368.25–697.95)	647.6 (226.28–693.37)	9/5	0.92
	TIMP-3	1881 (1024.22–2728.84)	987.8 (404.36–2938.49)	9/5	0.54

Quantitative data are presented as median (interquartile range, IQR). Comparisons between groups (<40 vs >40 years) were performed using the nonparametric Mann-Whitney U test. Matrix metalloproteinase (MMP) and tissue inhibitor of metalloproteinases (TIMP) were considered significant. *P* values < 0.05 were considered significant.

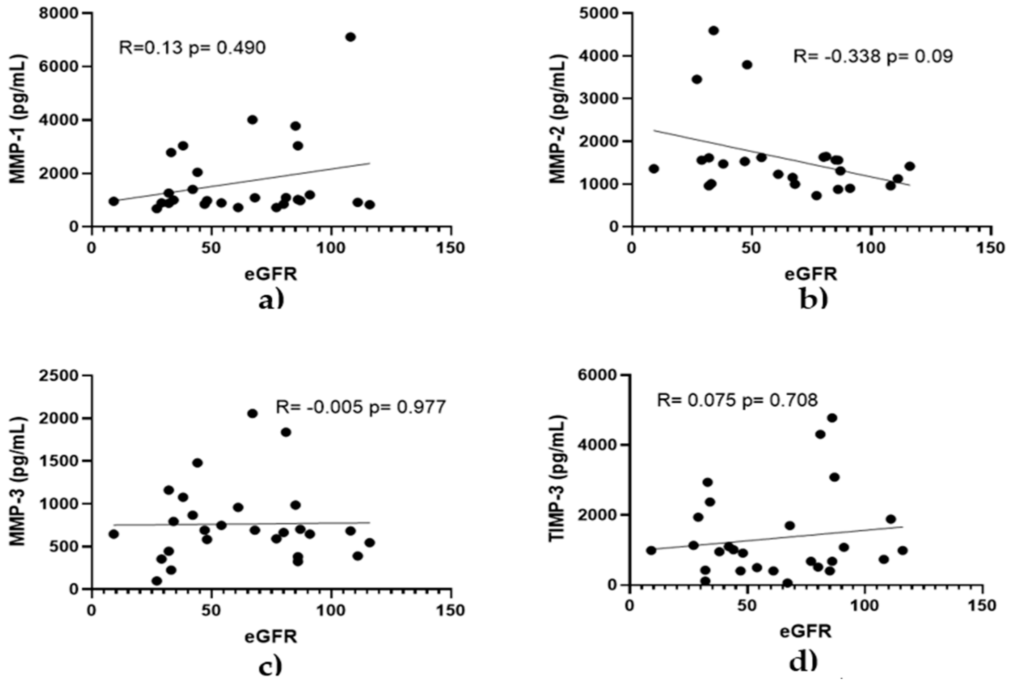

Figure S1: Correlation between the levels of (a) MMP-1, (b) MMP-2, (c) MMP-3 and (d) TIMP-3, all expressed in pg/mL and estimated glomerular filtration rate (eGFR), analyzed using Spearman's correlation coefficient (R). *P* values < 0.05 were considered significant.
